# Systemic therapy for psoriasis and the risk of cutaneous infections

**DOI:** 10.1111/1346-8138.17245

**Published:** 2024-04-25

**Authors:** Yuko Higashi, Shinichi Imafuku, Noriko Tsuruta, Kenta Murotani, Western Japan Inflammatory Skin Disease Research Group

**Affiliations:** ^1^ Department of Dermatology Kagoshima University Graduate School of Medical and Dental Sciences Kagoshima Japan; ^2^ Department of Dermatology Fukuoka University Faculty of Medicine Fukuoka Japan; ^3^ Division of Dermatology Kitakyushu City Yahata Hospital Kitakyusyu Japan; ^4^ Biostatistics Center Kurume University Kurume Japan

**Keywords:** cellulitis, herpes zoster, infections, oral candidiasis, psoriasis

## Abstract

Systemic treatments are important for patients with moderate‐to‐severe psoriasis; however, they may occasionally cause adverse infectious events. Although the risk of severe infections with psoriatic treatments is well established, little is known about cutaneous infections. Therefore, we studied the frequency of cutaneous infections in patients with psoriasis who underwent biologic treatment. A total of 878 patients (237 females and 641 males) were analyzed in this follow‐up survey conducted in 2020 and based on the Western Japan Psoriasis Registry. The observed skin phenotypes were psoriasis vulgaris (83.3%), pustular psoriasis (7.5%), and psoriatic arthritis (28.9%). The most frequently prescribed systemic drug was apremilast (11.3%), followed by ixekizumab (11.0%), risankizumab (10.9%), and secukinumab (10.4%). The incidence of cutaneous bacterial infections was 12 (1.37% of the total patients), with cellulitis being the most common (8/12, 67%). The incidence of viral infections was 11 (1.25%) including the most common, herpes zoster (9/11, 82%); and that of fungal infections was 45 (5.13%) including 33 (73%) and seven (16%) patients with trichophytosis and oral candidiasis, respectively. Multivariate analysis revealed that cutaneous bacterial infections were frequently observed in patients receiving tumor necrosis factor‐α (odds raio [OR] 9.917, 95% confidence interval [CI] 2.069–47.572, *p* = 0.004) and interleukin (IL)‐17 (OR 10.798, 95% CI 2.35–49.616, *p* = 0.002) inhibitor treatments. A history of otitis media and treatment with oral medications (OR 4.50, 95% CI 1.281–15.804, *p* = 0.019 and OR 3.80, 95% CI 1.141–12.679, *p* = 0.03 respectively) were associated with a higher ORs for cutaneous viral infections. Furthermore, age and use of IL‐17 inhibitors were associated with elevated ORs for fungal infections. In conclusion, our study reveals that systemic therapies may increase the risk of cutaneous viral infections. Therefore, dermatologists should exercise caution in this regard.

## 1 INTRODUCTION

Psoriasis is a chronic, immune‐mediated disease that affects approximately 125 million people worldwide,^1^ with approximately 2500 new cases in Japan annually.^2^ Systemic therapies with biologics, oral agents, and phototherapy are the mainstay of treatment for patients with moderate‐to‐severe psoriasis.^1^, ^3^ They may be used for localized diseases involving specific areas, such as the scalp, palms, soles, and genitals, or recalcitrant local psoriasis unresponsive to topical therapies.^1^ Biologics inhibit cytokines, such as tumor necrosis factor (TNF)‐α, interleukin (IL)‐17, IL‐23, and IL‐36. Oral treatments include conventional agents, such as methotrexate, acitretin or etretinate, and cyclosporine, and newly developed small molecules including phosphodiesterase 4, Janus kinase (JAK), and tyrosine kinase 2 inhibitors. The overshoot of the IL‐23‐IL‐17 axis inflammation is regarded as the pathophysiology of psoriasis.^1^ Moreover, IL‐17A and IL‐17F play important roles in the host defense against bacterial and fungal infections.^3^ Systemic treatments for psoriasis affect the host immune system and can cause various infections as side effects. Several reports exist on the risk of severe infections with the use of systemic therapies for psoriasis, which can lead to hospitalization.^4^, ^5^, ^6^, ^7^, ^8^ However, only limited data are available on the risk of cutaneous infections in patients receiving systemic therapy for psoriasis.^9^, ^10^

The Western Japan Psoriasis Registry (WJPR) is managed by independent dermatologists specializing in psoriasis medicine in western Japan. It was established in 2019,^11^ and has enrolled over 2000 patients with psoriasis. The WJPR is updated annually with newly occurring events including cutaneous infections. Additionally, it has revealed that genetics play a larger role in psoriatic arthritis development than in psoriasis vulgaris development.^12^ Biologics exhibit differences in their tendency to lose efficacy. The factors that negatively impact the survival rate of biologics include the history of biologics use, obesity, and psoriatic arthritis.^13^ Our previous analysis revealed a significantly greater number of female patients, lower mean body mass index (BMI), and a lower ratio of habitual drinkers in pustular psoriasis than in other psoriasis subtypes.^14^ None of the 29 human T‐lymphotropic virus type 1 carrier patients who received biologics experienced adult T‐cell leukemia/lymphoma.^15^

Therefore, using the WJPR update data, our novel study revealed the incidence of cutaneous infections during systemic treatments of psoriasis to provide useful information regarding drug selection, precautions, patient education, and infection control during psoriasis treatment in Japan.

## 2 METHODS

### 2.1 Participants

The WJPR is a multi‐institutional registry operated by 31 facilities in western Japan including university hospitals, community hospitals, and clinics located in the region.^16^ Appendix presents the names and affiliations of the physicians who contributed to this study. The WJPR enrolls patients with psoriasis whose current or previous systemic intervention was initiated after January 2010. A physician was responsible for conducting a yearly follow‐up survey for as long as the patient reported for follow‐up. We collected data regarding age, sex, BMI, psoriasis phenotype, body surface area (BSA), psoriasis severity, smoking status, drinking habits, comorbidities, systemic therapy, and cutaneous infections from all patients. The follow‐up survey commenced in 2020, and data were collected between September and December each year. We collected data on psoriasis severity, BSA, the incidence of malignancy, the incidence of infections, and all systemic anti‐psoriatic medications used during the survey year. Furthermore, this study analyzed data from 2020, and multivariate and univariate analyses were performed on these data. The Institutional Review Board of Fukuoka University Faculty of Medicine approved this study protocol (approval number: U19‐03‐004), and all patients provided written informed consent.

### 2.2 Statistical analyses

Continuous variables are summarized as median and interquartile range (IQR), while categorical variables are presented as frequencies and proportions. This study examined the presence or absence of bacterial, viral, and fungal infections as dependent variables. To explore the factors associated with each dependent variable, logistic regression analysis was conducted using patient characteristics, medical history, and medication use as independent variables. However, performing an adjusted analysis with several variables was challenging because of the limited number of events for the dependent variables in this study. Therefore, multivariate analysis was performed following a specific procedure. First, a univariate logistic regression analysis was employed to extract variables with a *p*‐value of ≤0.1. Subsequently, the variable selection was performed using a stepwise method for the extracted variables. Odds ratios (ORs), 95% confidence intervals (CIs), and *p*‐values were calculated for selected variables. This study was not adjusted for multiplicity because of its exploratory nature. All statistical analyses were performed using SAS 9.4 software (SAS Institute Inc.) Statistical significance was set at *p* < 0.05.

## 3 RESULTS

### 3.1 Patient characteristics

A total of 878 patients (237 females and 641 males) were enrolled in this survey, and their annual update data were collected and analyzed (Table 1). The median patient age and BMI were 59 (IQR 49–71) years and 24.78 kg/m,^2^ respectively. Additionally, the observed phenotypes were psoriasis vulgaris (83.3%), pustular psoriasis (7.5%), and psoriatic arthritis (28.9%). At enrollment, 70.5% of patients had an affected BSA of ≤3% and 67.0% had an overall Physician Global Assessment rating of 0 or 1. The prevalence of current and past smoking statuses was 32.5% and 34.4%, respectively. Habitual alcohol intake was 43.1%. The most common comorbidity was dental disease (39.4%), followed by hypertension (35.0%), allergic rhinitis (15.9%), diabetes (13.2%), and sinusitis (13.1%). Furthermore, the most frequently used systemic medication was apremilast (11.3% of the total cohort), followed by ixekizumab (11.0%), risankizumab (10.9%), secukinumab (10.4%), brodalumab (9.9%), ustekinumab (7.5%), guselkumab (7.3%), methotrexate (5.75%), etretinate (5.45%), adalimumab (4.9%), infliximab (3.8%), cyclosporin (2.3%), certolizumab pegol (0.9%), and bimekizumab (0.5%).

**TABLE 1:** Patient characteristics.

Characteristics	*n*	
Age, years, median (IQR)	59	(49.00–71.00)
Age at psoriasis onset (years), median (IQR)	38	(25.00–50.00)
Sex, *n* (%)		
Female	237	(27.0)
Male	641	(73.0)
Height (cm), mean	165.38	
BMI (kg/m^2^), mean	24.78	
Psoriasis phenotype, *n* (%)		
Psoriasis vulgaris	731	(83.3)
Psoriatic arthritis	254	(28.9)
Pustular psoriasis	66	(7.5)
BSA, *n* (%)		
≤3%	618	(70.5)
>3%, ≤10%	156	(17.8)
>10%, ≤ 20%	46	(5.2)
>20%	19	(2.2)
N/A	38	(4.3)
Psoriasis severity (PGA), *n* (%)		
0: clear	249	(28.7)
1: nearly clear	333	(38.3)
2: mild	175	(20.1)
3: moderate	60	(6.9)
4: severe	13	(1.5)
N/A	39	(4.5)
Arthralgia, n (%)	265	(30.2)
Smoking, *n* (%)		
Former smoker	301	(34.4)
Current smoker	285	(32.5)
Never smoked	290	(33.1)
Habitual drinking, *n* (%)		
Drinker	378	(43.1)
Light drinker	383	(43.7)
Former drinker	116	(13.2)
Comorbidities, *n* (%)		
Hypertension	307	(35.0)
Diabetes	116	(13.2)
Fatty liver	100	(11.4)
Hyperuricemia	58	(6.6)
Cardiovascular disease	33	(3.8)
Cerebrovascular disease	24	(2.7)
Cancer	69	(7.9)
Thyroid disease	38	(4.3)
Atopic dermatitis	48	(5.5)
Allergic rhinitis	140	(15.9)
Asthma	80	(9.1)
Dental diseases	346	(39.4)
Otitis media	97	(11.0)
Sinusitis	115	(13.1)
Tonsillitis	83	(9.5)
Tonsillectomy	54	(6.2)
Systemic therapy, *n* (%)		
Oral medicines	227	(25.9)
Apremilast	100	(11.4)
Cyclosporin	20	(2.3)
Deucravacitinib	3	(0.3)
Etretinate	50	(5.7)
Methotrexate	54	(6.2)
TNF‐α inhibitors	88	(10.0)
Adalimumab	44	(5.0)
Certolizumab Pegol	8	(0.9)
Infliximab	36	(4.1)
IL‐17 inhibitors	290	(33.0)
Bimekizumab	4	(0.5)
Brodalumab	89	(10.1)
Ixekizumab	100	(11.4)
Secukinumab	97	(11.0)
IL‐23 inhibitors	238	(27.1)
Guselkumab	66	(7.5)
Risankizumab	96	(10.9)
Ustekinumab	68	(7.5)
Others	8	(0.9)

Abbreviations: BMI, body mass index; BSA, body surface area; IL, interleukin; IQR, interquartile range; N/A, not applicable; PGA, Physician Global Assessment; TNF, tumor necrosis factor.

### 3.2 Incidence of cutaneous infections

#### 3.2.1 Bacterial infection

Cutaneous bacterial infection occurred in 12 patients (1.37%) (Table 2). Cellulitis was the most common (8/12, 67%), followed by one case each of abscess, erythrasma, folliculitis, and infected epidermal cysts (1/12, 8.3%). Univariate analysis revealed that in cutaneous bacterial infections, patients with pustular psoriasis and systemic treatment had a significantly higher OR of 4.25 (95% CI of 1.123–16.09) than those with other psoriasis phenotypes (Table 3). Among systemic treatments, bacterial infections were more frequent in users of TNF‐α and IL‐17 inhibitors. Multivariate analysis revealed that cutaneous bacterial infections were frequently observed in patients treated with TNF‐α (OR 9.917, 95% CI 2.069–47.572, *p* = 0.004) and IL‐17 (OR 10.798, 95% CI 2.35–49.616, *p* = 0.002) inhibitors (Table 6). Cellulitis alone, the most frequently occurring infection, showed results similar to those of the analysis of all bacterial infections. (Table S1).

**TABLE 2:** Incidence of cutaneous infections.

Infections	*n*	(%)
Cutaneous bacterial infections	12	(1.37)
Cellulitis	8	(0.91)
Abscess	1	(0.11)
Erythrasma	1	(0.11)
Folliculitis	1	(0.11)
Infectious epidermal cyst	1	(0.11)
Cutaneous viral infections	11	(1.25)
Herpes zoster	9	(1.03)
Herpes simplex	1	(0.11)
Condyloma acuminatum	1	(0.11)
Cutaneous fungal infections	45	(5.13)
Trichophytosis	33	(3.76)
Oral candidiasis	7	(0.80)
Non‐oral candidiasis	5	(0.57)
Tinea versicolor	1	(0.11)
Malassezia folliculitis	1	(0.11)

**TABLE 3:** Cutaneous bacterial infections (univariate logistic regression).

Characteristics	(*n* = 878)	Bacterial infections (*n* = 12)	OR	95% CI	*p*
Age			0.993	0.955–1.032	0.7077
Age at psoriasis onset			0.984	0.949–1.019	0.3607
Sex					
Female	237	3	0.993	0.261–3.775	0.9918
Male	641	9	1		
Height			1.035	0.965–1.11	0.3386
BMI			0.879	0.742–1.042	0.1364
Psoriasis phenotype					
Psoriasis vulgaris	731	9	0.598	0.16–2.237	0.4453
Psoriatic arthritis	254	5	1.77	0.557–5.629	0.3335
Pustular psoriasis	66	3	**4.25**	1.123–16.093	**0.0332**
BSA					
≤3%	618	7	1		
>3%, ≤10%	156	4	2.649	0.738–9.505	0.135
>10%, ≤20%	46	0	Not estimated		
>20%	19	0	Not estimated		
N/A	38	1	2.721	0.319–23.191	0.3599
PGA					
0: clear	249	4	1		
1: Nearly clear	333	2	0.373	0.068–2.056	0.2577
2: mild	175	4	1.433	0.353–5.808	0.6146
3: moderate	60	0	Not estimated		
4: severe	13	0	Not estimated		
N/A	39	1	1.612	0.175–14.808	0.6731
Arthralgia	265		1.33	0.386–4.582	0.6515
Smoking					
Former smoker	301	3	0.963	0.193–4.81	0.9632
Current smoker	285	6	1.734	0.41–7.324	0.4542
Never smoked	290	3	1		
Habitual drinking					
Drinker	378	5	1.26	0.336–4.73	0.7319
Light drinker	383	5	1		
Former drinker	116	2	1.665	0.301–9.211	0.559
Comorbidities					
Hypertension	307	6	1.877	0.6–5.87	0.279
Diabetes	116	0	Not estimated		
Fatty liver	100	1	0.704	0.09–5.514	0.7385
Hyperuricemia	58	1	1.291	0.164–10.171	0.8085
Cardiovascular disease	33	0	Not estimated		
Cerebrovascular disease	24	0	Not estimated		
Cancer	69	0	Not estimated		
Thyroid disease	38	0	Not estimated		
Atopic dermatitis	48	2	3.567	0.759–16.749	0.1071
Allergic rhinoritis	140	3	1.774	0.474–6.635	0.3946
Asthma	80	3	3.416	0.906–12.882	0.0697
Dental diseases	346	4	0.766	0.229–2.564	0.6656
Otitis media	97	2	1.623	0.35–7.519	0.5357
Sinusitis	115	1	0.6	0.077–4.689	0.626
Tonsillitis	83	1	0.869	0.111–6.818	0.8939
Tonsillectomy	54	1	1.395	0.177–11.006	0.7524
Systemic treatment of psoriasis during cutaneous infections					
Oral medicines	227	5	2.274	0.714–7.241	0.1644
TNF‐α inhibitors	88	4	**4.78**	1.409–16.218	**0.0121**
IL‐17 inhibitors	290	10	**6.622**	1.779–24.655	**0.0048**
IL‐23 inhibitors	238	2	0.566	0.123–2.604	0.4652
Others	8	0	Not estimated		

*Note*: Bold values denote statistical significance at the *p* < 0.05 level.

Abbreviations: BMI, body mass index; BSA, body surface area; CI, confidence interval; IL, interleukin; N/A, not applicable; OR, odds ratio; PGA, Physician Global Assessment; TNF, tumor necrosis factor.

#### 3.2.2 Viral infections

Cutaneous viral infections occurred in 11 patients (1.25%); herpes zoster was the most common (9/11, 82%), followed by herpes simplex (1/11, 9%), and condyloma acuminatum (1/11, 9%) (Table 2). Univariate analysis indicated that a history of otitis media and systemic treatment were associated with cutaneous viral infections (Table 4). Patients who received oral medications had the highest incidence of viral infections among systemic treatments (OR 3.851, 95% CI 1.163–12.747). Multivariate analysis revealed that a history of otitis media and treatment with oral medication were associated with a higher OR (4.50, 95% CI 1.281–15.804, *p* = 0.019 and 3.80, 95% CI 1.141–12.679, *p* = 0.03, respectively) of cutaneous viral infections than other groups (Table 6). Univariate analysis of herpes zoster alone, the most frequent of the viral infections, showed results similar to those of the analysis of all viral infections (Table S2).

**TABLE 4:** Cutaneous viral infections (univariate logistic regression).

Characteristics	(*n* = 878)	Viral infections (*n* = 11)	OR	95% CI	*p*
Age			1.018	0.976–1.062	0.4108
Age at psoriasis onset			1.004	0.968–1.041	0.8279
Sex					
Female	237	3	0.993	0.261–3.775	0.9918
Male	641	8	1		
Height			0.968	0.915–1.024	0.2611
BMI			1.006	0.886–1.142	0.9262
Psoriasis phenotype					
Psoriasis vulgaris	731	9	0.904	0.193–4.226	0.8977
Psoriatic arthritis	254	5	2.068	0.626–6.839	0.2336
Pustular psoriasis	66	0	Not estimated		
BSA					
≤3%	618	5	1		
>3%, ≤10%	156	4	3.184	0.845–12.001	0.0871
>10%, ≤ 20%	46	1	2.75	0.314–24.055	0.3606
>20%	19	0	Not estimated		
N/A	38	1	3.27	0.372–28.715	0.2851
PGA					
0: clear	249	2	1		
1: Nearly clear	333	3	1.133	0.188–6.833	0.8916
2: mild	175	5	3.632	0.697–18.94	0.1258
3: moderate	60	0	Not estimated		
4: severe	13	0	Not estimated		
N/A	39	1	3.25	0.288–36.719	0.3407
Arthralgia	265				
Smoking					
Former smoker	301	3	0.407	0.104–1.589	0.1957
Current smoker	285	1	0.144	0.018–1.181	0.0711
Never smoked	290	7	1		
Habitual drinking					
Drinker	378	4	1		
Light drinker	383	4	1.005	0.25–4.05	0.994
Former drinker	116	3	2.52	0.556–11.43	0.2308
Comorbidities					
Hypertension	307	7	3.308	0.961–11.39	0.0579
Diabetes	116	3	2.502	0.654–9.571	0.1803
Fatty liver	100	0	Not estimated		
Hyperuricemia	58	1	1.421	0.179–11.297	0.7397
Cardiovascular disease	33	0	Not estimated		
Cerebrovascular disease	24	0	Not estimated		
Cancer	69	1	1.175	0.148–9.316	0.8786
Thyroid disease	38	1	2.243	0.28–17.989	0.4469
Atopic dermatitis	48	2	3.968	0.833–18.89	0.0834
Allergic rhinitis	140	4	3.071	0.887–10.635	0.0766
Asthma	80	1	0.997	0.126–7.894	0.9981
Dental diseases	346	6	1.86	0.563–6.142	0.3086
Otitis media	97	4	**4.757**	1.367–16.556	**0.0142**
Sinusitis	115	1	0.661	0.084–5.209	0.6939
Tonsillitis	83	2	2.157	0.458–10.153	0.3306
Tonsillectomy	54	0	Not estimated		
Systemic treatment of psoriasis during cutaneous infections					
Oral medicines	227	5	**3.851**	1.163–12.747	**0.0273**
TNF‐α inhibitors	88	3	2.072	0.44–9.747	0.3564
IL‐17 inhibitors	290	3	0.803	0.211–3.05	0.7473
IL‐23 inhibitors	238	2	0.63	0.135–2.939	0.5568
Others	8	0	Not estimated		

*Note*: Bold values denote statistical significance at the *p* < 0.05 level.

Abbreviations: BMI, body mass index; BSA, body surface area; CI, confidence interval; IL, interleukin; N/A, not applicable; OR, odds ratio; PGA, Physician Global Assessment; TNF, tumor necrosis factor. Bold values denote statistical significance at the p〈 0.05 level.

#### 3.2.3 Fungal infections

Cutaneous fungal infections occurred in 45 patients (5.13%), and trichophytosis was the most common (33/45, 73%), followed by oral candidiasis (7/45, 16%), non‐oral candidiasis (5/45, 11%), tinea versicolor (1/45, 2%), and Malassezia folliculitis (1/45, 2%) (Table 2). In cutaneous fungal infections, older age, history of asthma, and use of IL‐17 inhibitors had a significantly higher OR than others (1.034, 95% CI 1.011–1.058, *p* = 0.0032; 2.286, 95% CI 1.026–5.095, *p* = 0.0432; and 3.137, 95% CI 1.705–5.772, *p* = 0.0002, respectively) (Table 5). Multivariate analysis revealed that older age and use of IL‐17 inhibitors were associated with elevated ORs for fungal infections (Table 6).

**TABLE 5:** Cutaneous fungal infections (univariate logistic regression).

Characteristics	(*n* = 878)	Fungal infections (*n* = 45)	OR	95%CI	*p*
Age			**1.034**	1.011–1.058	**0.0032**
Age at psoriasis onset			1.014	0.997–1.032	0.1069
Sex					
Female	237	11	1		
Male	641	34	1.088	0.539–2.195	0.8141
Height			0.997	0.977–1.017	0.7438
BMI			0.962	0.896–1.034	0.298
Psoriasis phenotype					
Psoriasis vulgaris	731	36	0.794	0.374–1.686	0.5485
Psoriatic arthritis	254	16	1.38	0.736–2.587	0.3157
Pustular psoriasis	66	4	1.213	0.421–3.498	0.7204
BSA					
≤3%	618	31	1		
>3%, ≤10%	156	11	1.467	0.718–2.996	0.2934
>10%, ≤20%	46	3	0.899	0.208–3.889	0.887
>20%	19	0	Not estimated		
N/A	38	0	Not estimated		
PGA					
0: clear	249	12	1		
1: Nearly clear	333	18	1.139	0.538–2.411	0.7329
2: mild	175	10	1.197	0.505–2.835	0.6828
3: moderate	60	3	1.039	0.284–3.806	0.9534
4: severe	13	0	Not estimated		
N/A	39	0	Not estimated		
Arthralgia	265	15			
Smoking					
Former smoker	301	20	1.435	0.695–2.963	0.3284
Current smoker	285	12	0.868	0.382–1.972	0.7357
Never smoked	290	13	1		
Habitual drinking					
Drinker	378	24	1.915	0.96–3.82	0.0653
Light drinker	383	15	1		
Former drinker	116	6	1.556	0.577–4.19	0.3822
Comorbidities					
Hypertension	307	20	1.522	0.831–2.787	0.1737
Diabetes	116	10	1.96	0.943–4.073	0.0715
Fatty liver	100	2	0.349	0.083–1.463	0.1498
Hyperuricemia	58	2	0.645	0.152–2.733	0.5521
Cardiovascular disease	33	1	0.569	0.076–4.26	0.5829
Cerebrovascular disease	24	1	0.801	0.106–6.064	0.8295
Cancer	69	0	Not estimated		
Thyroid disease	38	1	0.489	0.066–3.647	0.4853
Atopic dermatitis	48	1	0.38	0.051–2.819	0.3443
Allergic rhinitis	140	10	1.545	0.747–3.197	0.241
Asthma	80	8	**2.286**	1.026–5.095	**0.0432**
Dental diseases	346	21	1.368	0.749–2.497	0.3078
Otitis media	97	3	0.562	0.171–1.847	0.3422
Sinusitis	115	6	1.022	0.423–2.471	0.9614
Tonsillitis	83	6	1.51	0.62–3.682	0.3642
Tonsillectomy	54	3	1.095	0.328–3.655	0.8824
Systemic treatment of psoriasis during cutaneous infections					
Oral medicines	227	14	1.151	0.583–2.271	0.6852
TNF‐α inhibitors	88	6	1.448	0.595–3.526	0.4148
IL‐17 inhibitors	290	30	**3.137**	1.705–5.772	**0.0002**
IL‐23 inhibitors	238	12	1.039	0.527–2.048	0.9119
Others	8	0	Not estimated		

*Note*: Bold values denote statistical significance at the *p* < 0.05 level.

Abbreviations: BMI, body mass index; BSA, body surface area; CI, confidence interval; IL, interleukin; N/A, not applicable; OR, odds ratio; PGA, Physician Global Assessment; TNF, tumor necrosis factor. Bold values denote statistical significance at the p〈 0.05 level.

**TABLE 6:** Multivariate logistic regression analysis.

	OR	95% CI	*p*
Cutaneous bacterial infections			
TNF‐α inhibitors	9.917	2.069–47.542	0.0041
IL‐17 inhibitors	10.798	2.35–49.616	0.0022
Cutaneous viral infections			
Otitis media	4.499	1.281–15.804	0.019
Oral medicines	3.804	1.141–12.679	0.0296
Cutaneous fungal infections			
Age	1.04	1.007–1.073	0.0003
IL‐17 inhibitors	3.291	1.744–6.209	0.0002

Abbreviations: CI, confidence interval; IL, interleukin; OR, odds ratio; TNF, tumor necrosis factor.

## 4 DISCUSSION

Dermatologists usually encounter various cutaneous infections in the treatmen of psoriasis. Modern therapeutics have enabled extended intervals between visits for as long as 3 months; therefore, capturing minor signs or symptoms is sometimes difficult until they become intolerable. Cutaneous infections usually modify the clinical appearance of psoriasis and mislead dermatologists into selecting inappropriate options. Our study demonstrated that patients with psoriasis receiving systemic therapy had a risk of cutaneous infections, and the incidence differed by pathogens and therapeutic modalities.

Severe infections that are life‐threatening or require hospitalization have been extensively investigated in patients with psoriasis. The Spanish Registry of Adverse Events for Biological Therapy in Dermatological Diseases (BIOBADADERM registry) suggested that the adjusted risk ratio of overall infection significantly increased in the groups treated with adalimumab + methotrexate, infliximab, cyclosporine, ustekinumab + methotrexate, and etanercept compared with those treated methotrexate alone.^17^ Additionally, the cohort study from the French National Health Data System indicated that the risk of severe infections was higher for new users of adalimumab or infliximab than for users of etanercept, whereas ustekinumab was associated with a lower risk of severe infections.^4^ Therefore, while several studies have reported that systemic therapy, particularly TNF inhibitors, increases the risk of severe infection, others have suggested that the risk of severe infections is independent of treatment.^5^, ^6^

Our results revealed that TNF inhibitors were associated with an increased risk of cutaneous bacterial infections; 67% were cellulitis. An early study showed that one of the 23 infliximab‐treated patients had cellulitis.^18^ More extensive studies that described the association between bacterial infections and psoriasis treatment did not discover an overall difference in bacterial infections according to the treatment strategies; however, subanalysis based on the type of severe infection indicated that users of acitretin had a significantly higher risk of cellulitis than users of methotrexate.^5^

Our study also revealed that IL‐17 inhibitors had a high frequency of bacterial infections. IL‐17A and IL‐17F play important roles in host defense against bacterial infections.^3^ Specifically, IL‐17 receptor signaling is required for neutrophil recruitment and host defense.^19^ Reports indicate that both IL‐17F and IL‐17A are required for protection against bacterial infection.^20^ Consequently, these mechanisms may have affected our results. Among the bacterial infections, cellulitis was the most common and important one. These results were similar to those of the analysis of all bacterial infections (Table S1).

In our study, herpes zoster was the most frequent cutaneous viral infection, and a history of otitis media and oral medication administration were associated with a high risk of herpes zoster onset. However, no previous reports have associated otitis media with an increased incidence of herpes zoster. The genetic backgrounds of otitis media and herpes zoster have been investigated using genome‐wide association analyses, and no correlation was found.^21^ Because of the low incidence of herpes zoster, the correlation could be a coincidence, and this relationship should be further investigated. Univariate analysis of herpes zoster alone showed results similar to those of the analysis of all viral infections (Table S2).

Our data revealed that oral medications, excluding TNF‐α, were significantly associated with cutaneous viral infections. Several reports have described the relationship between oral treatments for psoriasis and herpes zoster. Shalom et al. reported that methotrexate and cyclosporine were not associated with an increased risk of herpes zoster in a cohort study of 95 941 patients with psoriasis.^22^ Hagberg et al. investigated the incidence of herpes zoster in patients with psoriasis in the MarketScan data and found that the combination of corticosteroids with other drugs (disease‐modifying antirheumatic drugs and TNF inhibitors) showed a higher incidence than apremilast alone or IL‐17/23 inhibitors alone.^23^ In our study, oral medications with different actions were grouped, therefore, how significantly each drug affected our results remains unclear. Patients with biologic‐avoiding factors may have an increased risk of herpes zoster; however, more data are required to confirm this result.

Our study demonstrated that the incidence of cutaneous fungal infections was numerically higher than bacterial and viral infections. Fungal infections were found more in patients treated with IL‐17 inhibitors than in others. IL‐17A is well known to play an important role in fungal infections. Although reports examining the relationship between psoriasis treatment and onychomycosis are available, most of them are outdated. A systematic review indicated that the prevalence of onychomycosis in patients with psoriasis and a control group, was 18% and 9.1%, respectively.^24^ These studies were specifically published by 2003, when biologic treatment was not yet available. A study of 81 patients with psoriasis diagnosed with nail disorders stated that the prevalence of onychomycosis in patients with psoriasis receiving conventional and anti‐TNF‐α therapies was higher than that in those not receiving treatment.^25^ This was published in an era when IL‐17 inhibitors were not yet widely used. A newly developed IL‐17 A/F inhibitor, bimekizumab, is known to cause candida infection more frequently.^26^ Our results show that more than 5% of patients have fungal infections annually; therefore, dermatologists should be cautious, particularly when using IL‐17 inhibitors.

This study had some limitations. First, the low incidence of infections made it difficult to identify true contributing factors, particularly in viral infections. Second, all systemic medications used for psoriasis treatment within 1 year were surveyed; therefore, we could not precisely identify which was responsible for the infection.

In conclusion, patients on systemic therapies are at risk of experiencing cutaneous infections, particularly fungal infections, although modern systemic therapies are highly effective. Therefore, dermatologists should consider this possibility and provide appropriate precautions. Additional anti‐psoriatic drugs with novel mechanisms of action, such as a tyrosine kinase 2 inhibitor, are becoming available for clinical use. However, further studies should be conducted to obtain more concrete results after accumulating data by observing registered patients.

## CONFLICT OF INTEREST STATEMENT

The WJPR is run by a non‐profit organization, the Western Japan Inflammatory Skin Disease Research Group, and is supported by the funding support from the Japanese Society for Psoriasis Research, Amgen, Abbvie, Eisai, Taiho Yakuhin Kogyo, Kyowa Kirin, Maruho, and Sun Pharma.

Shinichi Imafuku is an Editorial Board member of the *Journal of Dermatology* and a co‐author of this article. To minimize bias, he was excluded from all editorial decision‐making related to the acceptance of this article for publication.

## Supporting information


Table S1.
Table S2.

## 1

Yuko Higashi (Department of Dermatology, Kagoshima University Graduate School of Medical and Dental Sciences, Kagoshima, Japan), Shinichi Imafuku (Department of Dermatology, Fukuoka University Faculty of Medicine, Fukuoka, Japan), Noriko Tsuruta (Department of Dermatology, Fukuoka University Faculty of Medicine, Fukuoka, Japan), Kenta Murotani (Biostatistics Center, Kurume University, Kurume, Japan), Kazuki Yamaguchi (Department of Dermatology, Fukuoka University Faculty of Medicine, Fukuoka, Japan), Kotaro Ito (Department of Dermatology, Fukuoka University Faculty of Medicine, Fukuoka, Japan), Takeshi Nakahara (Department of Dermatology, Graduate School of Medical Sciences, Kyushu University, Fukuoka, Japan), Eri Katayama (Department of Dermatology, Kurume University School of Medicine, Kurume, Japan), Chika Ohata (Department of Dermatology, Kurume University School of Medicine, Kurume, Japan), Bungo Ohyama (Department of Dermatology, Kurume University School of Medicine, Kurume, Japan), Etsuko Okada (Department of Dermatology, University of Occupational and Environmental Health, Kitakyushu, Japan), Natsuko Sasaki (Department of Dermatology, University of Occupational and Environmental Health, Kitakyushu, Japan), Maki Kuwashiro (Division of Dermatology, Department of Internal Medicine, Faculty of Medicine, Saga University, Saga, Japan), Aki Hashimoto (Division of Dermatology, Department of Internal Medicine, Faculty of Medicine, Saga University, Saga, Japan), Hiroyuki Murota (Department of Dermatology, Nagasaki University Graduate School of Biomedical Sciences, Nagasaki, Japan), Yuta Koike (Department of Dermatology, Nagasaki University Graduate School of Biomedical Sciences, Nagasaki, Japan), Yutaka Hatano (Department of Dermatology, Faculty of Medicine, Oita University, Yufu, Japan), Kanami Saito (Department of Dermatology, Faculty of Medicine, Oita University, Yufu, Japan), Kenzo Takahashi (Department of Dermatology, Graduate School of Medicine University of the Ryukyus, Nishihara, Japan), Takuya Miyagi (Department of Dermatology, Graduate School of Medicine University of the Ryukyus, Nishihara, Japan), Sakae Kaneko (Department of Dermatology, Shimane University Faculty of Medicine, Shimane, Japan), Masataka Ota (Department of Dermatology, Shimane University Faculty of Medicine, Shimane, Japan), Kazunari Sugita (Division of Dermatology, Department of Medicine of Sensory and Motor Organs, Faculty of Medicine, Tottori University, Yonago, Japan), Shin Morizane (Department of Dermatology, Okayama University Graduate School of Medicine, Dentistry and Pharmaceutical Sciences, Okayama, Japan), Kenta Ikeda (Department of Dermatology, Okayama University Graduate School of Medicine, Dentistry and Pharmaceutical Sciences, Okayama, Japan), Satoko Kikuchi (Department of Dermatology, Kyushu Central Hospital, Fukuoka, Japan), Kayo Harada (Department of Dermatology and Allergy, National Hospital Organization, Kyushu Medical Center, Fukuoka, Japan), Kentaro Yonekura (Department of Dermatology, Imamura General Hospital, Kagoshima, Japan), Tetsuji Yanase (Department of Dermatology, Hiroshima City North Medical Center Asa Citizens Hospital, Hiroshima, Japan), Yuki Matsuzaka (Department of Dermatology, Onomichi General Hospital, Onomichi, Japan), Fusako Okazaki (Department of Dermatology, Okayama City General Medicine Center, Okayama, Japan), Hiroshi Saruwatari (Saruwatari Dermatology Clinic, Kagoshima, Japan).
