# Supplementary material for: Systemic therapy for psoriasis and the risk of cutaneous infections
Source: J Dermatol. 2024 Apr 25;51(7):939–49. doi: 10.1111/1346-8138.17245 (PMC11483952; doi:10.1111/1346-8138.17245)
Supplement: Supplementary file 1 — Table S1. Table S2. [file JDE-51--s001.docx]

Supplemetary table 1: Cellulitis (univariate logistic regression)

| **Characteristics** | |  | **OR** |  | **95%CI** | ***P*-value** |
| --- | --- | --- | --- | --- | --- | --- |
| **Age** | |  | 1.019 |  | 0.969-1.071 | 0.4647 |
| **Age at psoriasis onset** | |  | 0.997 |  | 0.956-1.039 | 0.8778 |
| **Sex** | |  |  |  |  |  |
|  | Female |  | 1 |  |  |  |
|  | Male |  | 1.118 |  | 0.224-5.58 | 0.8915 |
| **Height** | |  | 1.01 |  | 0.935-1.09 | 0.8062 |
| **BMI** | |  | 0.957 |  | 0.807-1.134 | 0.6106 |
| **Psoriasis phenotype** | |  |  |  |  |  |
|  | Psoriasis vulgaris |  | 1.412 |  | 0.172-11.56 | 0.748 |
|  | Psoriatic arthritis |  | 0.818 |  | 0.164-4.077 | 0.8059 |
|  | pustular psoriasis |  | 4.198 |  | 0.83-21.221 | 0.0827 |
| **BSA** | |  |  |  |  |  |
|  | ≦3% |  | 1 |  |  |  |
|  | ＞3%, ≦10% |  | 2.971 |  | 0.658-13.412 | 0.1569 |
|  | ＞10%, ≦20% |  | not estimated | | |  |
|  | ＞20% |  | not estimated | | |  |
|  | N/A |  | 4.095 |  | 0.446-37.561 | 0.2125 |
| **PGA** | |  |  |  |  |  |
|  | 0: clear |  | 1 |  |  |  |
|  | 1: nearly clear |  | 0.753 |  | 0.105-5.383 | 0.7775 |
|  | 2: mild |  | 2.154 |  | 0.356-13.028 | 0.4043 |
|  | 3: moderate |  | not estimated | | |  |
|  | 4: severe |  | not estimated | | |  |
|  | N/A |  | 3.25 |  | 0.288-36.719 | 0.3407 |
| Arthralgia | |  | 1.396 |  | 0.331-5.883 | 0.6496 |
| Smorking | |  |  |  |  |  |
|  | Former smoker |  | 0.64 |  | 0.106-3.857 | 0.626 |
|  | Current smoker |  | 1.033 |  | 0.207-5.16 | 0.9688 |
|  | Never smoked |  | 1 |  |  |  |
| **Habitual drinking** | |  |  |  |  |  |
|  | Drinker |  | 1.344 |  | 0.299-6.047 | 0.6999 |
|  | Light drinker |  | 1 |  |  |  |
|  | Former drinker |  | 1.103 |  | 0.114-10.709 | 0.9325 |
| **Comorbidities** | |  |  |  |  |  |
|  | Hypertension |  | **5.671** |  | 1.138-28.269 | **0.0342** |
|  | Diabetes |  | not estimated | | |  |
|  | Fatty liver |  | 1.113 |  | 0.135-9.137 | 0.9209 |
|  | Hyperuricemia |  | 2.038 |  | 0.247-16.848 | 0.5087 |
|  | Cardiovascular disease |  | not estimated | | |  |
|  | Cerebrovascular disease |  | not estimated | | |  |
|  | Cancer |  | not estimated | | |  |
|  | Thyroid disease |  | not estimated | | |  |
|  | Atopic dermatitis |  | 2.502 |  | 0.302-20.753 | 0.3957 |
|  | Allergic rhinoritis |  | 0.752 |  | 0.092-6.155 | 0.7901 |
|  | Athema |  | not estimated | | |  |
|  | Dental diseases |  | 0.922 |  | 0.219-3.882 | 0.9117 |
|  | Otitis media |  | 2.719 |  | 0.541-13.664 | 0.2246 |
|  | Sinusitis |  | 0.947 |  | 0.115-7.771 | 0.9598 |
|  | Tonsillitis |  | 1.373 |  | 0.167-11.296 | 0.7682 |
|  | Tonsillectomy |  | 2.202 |  | 0.266-18.23 | 0.4641 |
| **Systemic treatment of psoriasis during cutaneous infections** | | | | | |  |
|  | Oral medicines |  | 1.898 |  | 0.45-8.008 | 0.3832 |
|  | TNF-α inhibitors |  | **5.689** |  | 1.336-24.233 | **0.0187** |
|  | IL-17 inhibitors |  | **6.561** |  | 1.316-32.713 | **0.0218** |
|  | IL-23 inhibitors |  | 0.95 |  | 0.19-4.74 | 0.95 |
|  | Others |  | not estimated | | |  |

Supplemetary table 2: Herpes zoster (univariate logistic regression)

| **Characteristics** | |  | **OR** |  | **95%CI** | ***P*-value** |
| --- | --- | --- | --- | --- | --- | --- |
| **Age** | |  | 1.028 |  | 0.979-1.078 | 0.2664 |
| **Age at psoriasis onset** | |  | 1.01 |  | 0.972-1.049 | 0.6127 |
| **Sex** | |  |  |  |  |  |
|  | Female |  | 1 |  |  |  |
|  | Male |  | 1.307 |  | 0.27-6.335 | 0.7398 |
| **Height** | |  | 0.973 |  | 0.913-1.037 | 0.3957 |
| **BMI** | |  | 0.984 |  | 0.847-1.143 | 0.8299 |
| **Psoriasis phenotype** | |  |  |  |  |  |
|  | Psoriasis vulgaris |  | 0.701 |  | 0.1443.408 | 0.6597 |
|  | Psoriatic arthritis |  | 1.981 |  | 0.5287.437 | 0.3112 |
|  | pustular psoriasis |  | not estimated | | |  |
| **BSA** | |  |  |  |  |  |
|  | ≦3% |  | 1 |  |  |  |
|  | ＞3%, ≦10% |  | **5.325** |  | 1.179-24.042 | **0.0297** |
|  | ＞10%, ≦20% |  | 4.598 |  | 0.469-45.127 | 0.1904 |
|  | ＞20% |  | not estimated | | |  |
|  | N/A |  | 5.468 |  | 0.555-53.859 | 0.1454 |
| **PGA** | |  |  |  |  |  |
|  | 0: clear |  | 1 |  |  |  |
|  | 1: nearly clear |  | 1.512 |  | 0.136-16.771 | 0.7362 |
|  | 2: mild |  | 7.294 |  | 0.845-62.989 | 0.0708 |
|  | 3: moderate |  | not estimated | | |  |
|  | 4: severe |  | not estimated | | |  |
|  | N/A |  | 6.526 |  | 0.4-106.549 | 0.188 |
| Arthralgia | |  |  |  |  |  |
| Smorking | |  |  |  |  |  |
|  | Former smoker |  | 0.574 |  | 0.136-2.423 | 0.4496 |
|  | Current smoker |  | 0.204 |  | 0.024-1.754 | 0.1474 |
|  | Never smoked |  | 1 |  |  |  |
| **Habitual drinking** | |  |  |  |  |  |
|  | Drinker |  | 2.022 |  | 0.368-11.104 | 0.418 |
|  | Light drinker |  | 1 |  |  |  |
|  | Former drinker |  | 5.068 |  | 0-836-30.709 | 0.0775 |
| **Comorbidities** | |  |  |  |  |  |
|  | Hypertension |  | 2.347 |  | 0.626-8.805 | 0.206 |
|  | Diabetes |  | 1.892 |  | 0.388-9.222 | 0.4299 |
|  | Fatty liver |  | not estimated | | |  |
|  | Hyperuricemia |  | 1.781 |  | 0.219-14.485 | 0.5895 |
|  | Cardiovascular disease |  | not estimated | | |  |
|  | Cerebrovascular disease |  | not estimated | | |  |
|  | Cancer |  | 1.472 |  | 0.181-11.947 | 0.7172 |
|  | Thyroid disease |  | 2.811 |  | 0.343-23.064 | 0.3358 |
|  | Atopic dermatitis |  | 2.186 |  | 0.268-17.844 | 0.4653 |
|  | Allergic rhinoritis |  | 1.513 |  | 0.311-7.362 | 0.6077 |
|  | Athema |  | not estimated | | |  |
|  | Dental diseases |  | 1.935 |  | 0.516-7.259 | 0.3275 |
|  | Otitis media |  | **4.122** |  | 1.01416.756 | **0.0477** |
|  | Sinusitis |  | 0.828 |  | 0.103-6.681 | 0.8593 |
|  | Tonsillitis |  | 1.2 |  | 0.148-9.712 | 0.8644 |
|  | Tonsillectomy |  | not estimated | | |  |
| **Systemic treatment of psoriasis during cutaneous infections** | | | | | |  |
|  | Oral medicines |  | **6.438** |  | 1.596-25.968 | **0.0089** |
|  | TNF-α inhibitors |  | 1.153 |  | 0.142-9.33 | 0.8938 |
|  | IL-17 inhibitors |  | 1.074 |  | 0.267-4.327 | 0.9197 |
|  | IL-23 inhibitors |  | 0.354 |  | 0.044-2.842 | 0.3283 |
|  | Others |  | not estimated | | |  |
